# ARF: the most misunderstood GTPase I ever knew - why study ARF GAPs

**DOI:** 10.3389/fmolb.2025.1668286

**Published:** 2025-10-13

**Authors:** Rachel E. Turn, Joel Bryan Dacks, Eric M. Rosenberg, Olivier Soubias, John K. Northup, Paul A. Randazzo

**Affiliations:** ^1^ Department of Microbiology and Immunology, Stanford University School of Medicine, Stanford, CA, United States; ^2^ Department of Medicine, University of Alberta, Edmonton, AB, Canada; ^3^ Laboratory of Cellular and Molecular Biology, National Cancer Institute, Bethesda, MD, United States; ^4^ National Cancer Institute, Bethesda, MD, United States

**Keywords:** ADP-ribosylation factor, ARF, GTPase, GTPase-activating protein, G protein, RAS, P loop NTPase

## Abstract

ADP-ribosylation factors (ARFs) are GTP-binding proteins that were discovered in the early 1980s, shortly after heterotrimeric GTP-binding proteins (G proteins) and nearly simultaneously with RAS. G proteins formed the basis for the signaling paradigm that has been broadly applied to GTPases, including both RAS and ARF. In this paradigm, GTP-binding proteins act as switches. When converted from the GDP-bound form to the GTP-bound form, GTPases bind effector proteins to transduce a signal. This paradigm is consistent, at least in part, with RAS function as RAS•GTP activates effectors to drive cellular responses such as proliferation. ARF, on the other hand, functions outside this paradigm, at least in its first discovered physiological role: regulation of membrane traffic. Nevertheless, ARFs are often generalized as “on” and “off” switches controlling signaling pathways. In this study, we (i) briefly describe the history of the discoveries of three families of GTPases to provide an understanding of the genesis of the G-protein signaling model, (ii) enumerate some key differences between ARFs, RAS, and G proteins (which better fit the paradigm of molecular switches), and (iii) describe an alternate model for ARFs, in which their cycling between GTP binding and hydrolysis mediates cellular activities, rather than ARFs acting as mediators in a signaling cascade. Furthermore, we highlight the key role of GTPase-activating proteins (GAPs) as integral to ARF function.

## 1 Prologue. ARF GTPases: current understanding and framing an argument for paradigm change

The ADP-ribosylation factor (ARF) family of small GTPases is an ancient group of proteins, with origins traceable to the last eukaryotic common ancestor (LECA) and earlier (Vargová et al., 2025). This critical family of 30 low-molecular-weight proteins in humans is known for the vital roles that they play in diverse cellular functions, ranging from cell division, cytoskeleton rearrangement, lipid metabolism, tubulin folding, mitochondrial dynamics, primary cilia formation, vesicular traffic, and more. Like other small GTPases, ARFs are often referred to as “molecular switches,” positioned at the crossroads of diverse signaling pathways and capable of turning pathways “on” and “off” based on their nucleotide-binding state. Understanding the molecular mechanisms by which ARF GTPases mediate essential cellular processes remains an elusive yet critical field of study, with implications both for a fundamental understanding of cellular mechanisms and for human health.

ARF, discovered in 1984, owes its name to its pathophysiologic activity as a cofactor for cholera toxin-catalyzed ADP ribosylation used to identify G_s_ (Kahn and Gilman, 1984), hence the term ADP-ribosylation factor. In 1986, ARF was found to bind GTP (Kahn and Gilman, 1986), and in the early 1990s, physiologic functions in membrane traffic and actin remodeling were discovered (Donaldson et al., 1992; Radhakrishna et al., 1999; Radhakrishna et al., 1996; Serafini et al., 1991). For many years, ARFs were assumed to function as signaling GTPases such as G proteins because i) ARFs are GTP-binding proteins that were discovered nearly simultaneously with RAS GTPases and shortly after the signaling function of G proteins was defined, and, perhaps, ii) they were discovered as a by-product of research on heterotrimeric G proteins.

**FIGURE 1:**
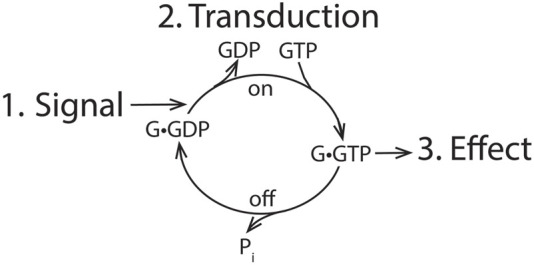
General outline of signaling. An extracellular signal is transduced into a cellular effect by the action of a switchable transduction mechanism. For GTPases, the system is turned to an “on” position by exchange of GDP for GTP and switched to an “off” position by hydrolyzing GTP.

The standard model for signaling encompasses three elements, as illustrated in Figure 1: the signal, the cellular effect, and a means of transducing the signal to achieve the effect. The transduction mechanism functions as a switch, going from an “on” state to an “off” state, achieved in G proteins by a cycle of GTP binding for activation and hydrolysis for GTPase inactivation. G proteins and RAS have intrinsic GTPase activity to switch off the signal, and GTPase-activating proteins (GAPs for the RAS family and RGS for G proteins) accelerate GTP hydrolysis either to terminate or block the signal. Previous studies identified the first physiological role of ARFs in membrane traffic, which deviates from the signaling paradigm. Nevertheless, exploration of ARF function continues to be biased against the possibility that ARFs are direct mediators of cell function.

In this study, we question the validity of the signaling paradigm for the founding members of the ARF family: ARF1 and ARF3 (∼97% identical) in humans and the closely related ARF4 and ARF5 (>80% identical). We first describe the discovery of heterotrimeric G proteins and other elements of the signaling paradigm, providing a clear basis for comparison with both RAS and ARF. Later, we describe how the paradigm is inadequate for explaining ARF function. To identify other functional models, we consider that ARF, RAS, and G proteins are part of a larger family of P loop nucleoside triphosphatases (NTPases) defined by the Walker A motif of GxxxxGKS/T, which is important for catalyzing phospho transfers (Leipe et al., 2003; Maggiolo et al., 2023; Walker et al., 1982; Zheng et al., 2024). Many have functions apart from signaling, including macromolecular structure assembly, proofreading, protein synthesis, or movement (i.e., motor proteins). Some might be more relevant models for ARF function than signaling GTPases. One ARF family member, ARL2, has a well-established function outside of the signaling paradigm (Francis et al., 2017a; Francis et al., 2017b). In alternate models that we are considering, the function of ARFs is integrated with GTPase-activating proteins (GAPs). ARF GAPs were identified based on their ability to induce GTP hydrolysis when bound to ARF. Because GAPs for signaling GTPases are thought to limit signaling by reducing GTP-bound GTPase levels, it has been widely assumed that ARF GAPs serve a similar function. In sharp contrast to this view, we hypothesize that ARF GAPs mediate ARF function rather than control ARF•GTP levels. Finally, we discuss future directions for the field and critical questions to be addressed concerning the mechanisms by which ARFs and ARF GAPs facilitate essential cellular processes.

## 2 The story of ARF

### 2.1 Chapter 1. discovery of ARF as a by-product of elucidating β-adrenergic receptor signaling explains the focus of the signaling paradigm for ARF

The identification of the ARF family was an unexpected observation made during efforts to establish the biochemistry of β-adrenergic receptor signaling. Our current molecular understanding of cell-surface receptor-initiated biochemical responses to extracellular signals builds on more than a century of physiological and pharmacological studies of autonomic and endocrine hormone-stimulated tissue responses (Gilman, 1987; Kahn, 2014; Lorente et al., 2025; Rohrer and Kobilka, 1998; Wess, 1997). The breakthrough physiological chemistry studies that eventually led to the discovery of ARF involved the mediation of β-adrenergic receptor signaling by cAMP, a second messenger compound generated by the intracellular enzyme adenyl cyclase (Murad et al., 1962; Rall and Sutherland, 1958; 1962; 1959; Sutherland and Rall, 1958; 1960; Sutherland et al., 1962). This was followed by the identification of literally hundreds of endocrine, autocoid, and other hormonal and humoral agents as activators of adenyl cyclase in mammalian tissues (Birnbaumer, 1990; Borrelli et al., 1992; Chandra Jena et al., 2024; Choi et al., 1993; Hanoune and Defer, 2001; McCudden et al., 2005). Of particular importance was groundbreaking work from Martin Rodbell’s laboratory, demonstrating a requirement for GTP in adenyl cyclase activation by glucagon and beta-adrenergic receptors (Harwood et al., 1973; Lad et al., 1980; Rodbell, 1980; Rodbell et al., 1971; Rodbell et al., 1975; Schramm and Rodbell, 1975; Yamamura and Rodbell, 1976). Their work led to the hypothesis that there is a GTP-binding protein (which we will now refer to as “Gs” for G-protein stimulator of adenyl cyclase) that transduces receptor activation by ligand into adenyl cyclase activation. A major line of evidence that drove the discovery of the Gs protein was the irreversible activation of adenyl cyclase by the entero-toxin secreted by *Vibrio cholera* (cholera toxin), which correlated with the ADP ribosylation of a 44 kDa protein in multiple cell types (Gill and Meren, 1978). Interestingly, later studies discovered that the S49 lymphoma cell line, termed cyc-, lacked beta-adrenergic receptor-mediated activation of adenylyl cyclase (Bourne et al., 1975a; Bourne et al., 1975b; Daniel et al., 1973) and did not contain the cholera toxin-labeled protein (Haga et al., 1977). The ability to restore beta-adrenergic activation of adenyl cyclase in detergent extracts of membranes from cyc- S49 cells with deficient adenyl cyclase activity (Ross and Gilman, 1977; Ross et al., 1978) provided a quantitative biochemical assay for Gs protein activity. These functional assays were subsequently used to aid in the purification of the Gs protein from detergent extracts of liver membranes (Northup et al., 1980), the original source for identifying epinephrine-induced cyclic AMP.

Two unexpected and immensely important findings came from the purification of the Gs protein. First, it was purified not as a single polypeptide chain (the single-gene product mutant in S49 cyc-), but as a heterotrimeric protein with a GTP-binding 44 kDa α-subunit affiliated tightly to 35 kDa β and 8 kDa γ polypeptides (Northup et al., 1982; Northup et al., 1983a; Northup et al., 1983b; Northup et al., 1980). The latter “βγ” subunits are so tightly complexed that they have not been successfully resolved without denaturing the β-chain. The βγ dimer was present in large excess of the 44 kDa protein in liver extracts (Northup et al., 1983b), a finding that led to the identification of multiple 40 kDa Gi α-subunits associated with the same βγ subunits (“i” for inhibiting adenyl cyclase) (Bokoch et al., 1983; Bokoch et al., 1984; Katada, et al., 1984a; Katada et al., 1984b) and the α-q/11/14 phospholipase C-activating G-proteins, which bound to a column of immobilized βγ subunits (Blank et al., 1991; Smrcka et al., 1991; Wange et al., 1991). This initial purification would set the stage for later work involving cloning of multiple additional related gene products for α, β, and γ subunit chains, now identified as the heterotrimeric GTP-binding “G-protein” family.

**FIGURE 2:**
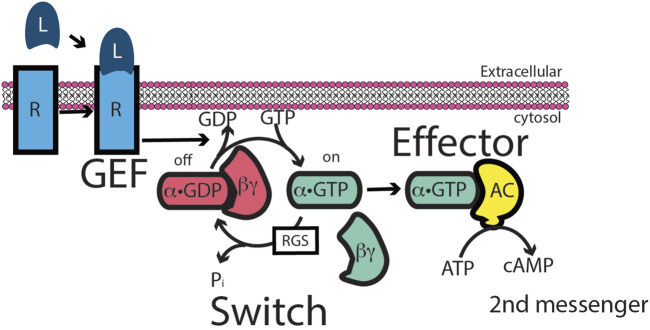
Heterotrimeric G-protein signaling paradigm. The paradigm was based on the intracellular activation of adenylyl cyclase in response to an extracellular ligand. In the paradigm, the extracellular ligand binds to a G-protein-coupled receptor (GPCR). The liganded GPCR acts as an exchange factor for Gs, a heterotrimer of α, β, and γ subunits, by inducing α dissociation from βγ and dissociation of GDP from α. α is then free to bind GTP. α•GTP binds to adenyl cyclase, stimulating catalysis to convert ATP to the second messenger cAMP. There are two points of signal amplification in the paradigm. First, liganded GPCR acts catalytically to convert multiple molecules of Gs to α-GTP. Second, adenyl cyclase is an enzyme, generating multiple molecules of cAMP.

Numerous studies of G-protein-coupled receptor “GPCR” signaling have led to a “canonical” G-protein signaling model (see cartoon) (Figure 2). Extracellular ligand recognition by a GPCR leads to the Gα subunit of the receptor-bound G-protein exchanging GDP for GTP; this leads to the consequent dissociation of the G-protein from the GPCR. The GTP-bound activated Gα subunit then binds to and activates its target effector enzyme, leading to the accumulation of an intracellular second messenger or another regulated event. Because in almost all instances, the activated lifetime of the ligand-bound GPCR vastly exceeds the time required to affect the GDP–GTP exchange, this initial step in GPCR signaling is highly amplified, with the accumulation of hundreds of GTP-bound activated Gα proteins per receptor–ligand binding event (Gilman, 1987; Jiang et al., 2022; Liu et al., 2024). Although initially identified and purified based on β-adrenergic receptor activation of the Gs protein, the most thoroughly studied and best understood GPCR is rhodopsin. Rhodopsin and its affiliated G-protein Gt activate a powerfully, catalytically active, retinal-specific cyclic GMP phosphodiesterase. In vertebrate photoreceptors, a single photon (i.e., one activated rhodopsin molecule) is sufficient to trigger electrical signaling to downstream neurons because of this amplified cascade, in which a single photon capture activates 300 Gt α molecules. We will visit this model later when comparing it with the mechanism of ARF function.

The second finding from purifying Gs was that ADP ribosylation by cholera toxin was lost from partially purified Gs. A component from membranes (present in cyc- membranes) restored labeling (Schleifer et al., 1982). The success of the G_s_ reconstitution assay provided the framework for developing an assay to identify and purify the ∼20 kDa cholera toxin-sensitive protein ARF (Kahn and Gilman, 1984; Schleifer et al., 1982). This ARF assay required the inclusion of GTP originally because of the assumption that it was bound to the Gs substrate. Only later was it revealed that ARF is itself a regulatory GTPase whose cofactor/ARF activity is dependent on GTP binding (Kahn and Gilman, 1986). Approximately the same size as the newly discovered RAS proteins, it was predicted that they were one and the same protein—an idea that was quickly dismissed when RAS was found to have no capacity to act as a cofactor for cholera toxin for ADP-ribosylation of Gs. Nevertheless, with the importance of RAS to cancer biology (still the most commonly found human oncogene) (Asimgil et al., 2022; Campbell and Der, 2004; Der, 1989; Dillon et al., 2021; Singhal et al., 2024; Spencer-Smith and Morrison, 2024), RAS became the paradigm for small, monomeric, regulatory GTPases, and ARF was assumed to have a similar signaling function.

**FIGURE 3:**
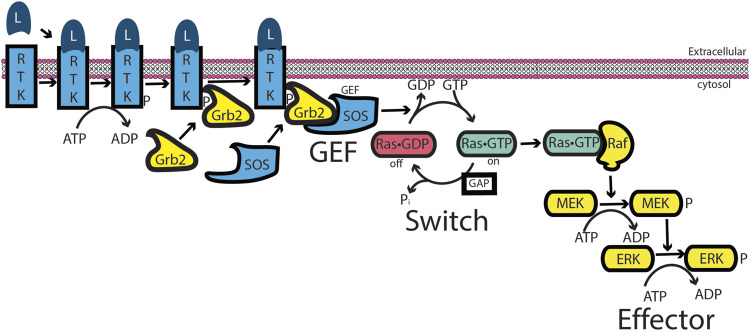
RAS signaling. Similar to G-protein signaling, an extracellular ligand binds to a receptor—here, a receptor tyrosine kinase (RTK). RTK autophosphorylates its cytoplasmic tail. An adaptor protein, Grb2, binds to the phosphorylation site and binds to an exchange factor, SOS, thereby recruiting SOS to the membrane in which RAS resides. For low-molecular-weight GTPases, the exchange factors function by a ping-pong bi-bi mechanism, driving RAS toward the GTP-bound form (Goody, 2014; Goody and Hofmann-Goody, 2002; Northup et al., 2012; Randazzo et al., 2013). RAS•GTP binds to and activates RAF, a serine/threonine kinase that initiates a cascade of protein phosphorylations, leading to a biological response.

Models for RAS, a monomeric or “small GTP-binding protein,” incorporated some elements of G-protein signaling. In this model (Khosravi-Far and Der, 1994) (Figure 3), the signal is initiated by an extracellular ligand binding to a receptor tyrosine kinase (RTK), resulting in its autophosphorylation. An adaptor protein (Grb2 in the illustration) binds simultaneously to the phosphorylated receptor and a guanine nucleotide exchange factor (SOS in the illustration) for RAS. This complex acts in the same capacity as a liganded GPCR. RAS•GTP then binds and activates the first in a series of protein kinases, termed a “phosphorylation cascade,” leading to a biological response. Intrinsic GTPase activity and GAP-mediated GTPase activity terminate or inhibit the signal by returning RAS to its inactive, GDP-bound state. This model mirrors that of G-protein signaling, in which an extracellular ligand functions through a transmembrane receptor that transduces an intracellular signal by increasing the GTP-bound form of RAS, thereby promoting binding to and activation of an enzyme referred to as the effector. As with other low-molecular-weight GTPases discovered around this time, ARF was considered to be part of the RAS superfamily and to function in a similar capacity in signaling. This family assignment for ARF was later found to be simplistic, if not erroneous (see next section), but many outside the field continue to misconstrue ARF as a member of the RAS superfamily.

### 2.2 Chapter 2. the ARF family of proteins is not part of the RAS superfamily of signaling GTPases, nor does ARF function like RAS

ARF is distinct from other regulatory low-molecular-weight GTP-binding proteins in at least five respects.

First, although ARFs are often referred to as members of the RAS superfamily, they constitute a distinct family with a separate evolutionary origin from that of RAS. The larger ARF family proper in mammals includes 6 ARFs, 20 ARF-like (ARL) proteins, ARFRP1, 2 SARs, and TRIM23. ARFs, SAR, and ARLs are found across eukaryotes, and the last eukaryotic common ancestor is inferred to have possessed 16 ARF members (Vargová et al., 2025; Vargová et al., 2021). This includes two ARFs, nine ARLs, two SARs, and ARFRP1. Notably, all these protein families have recently been shown to have evolved from a prokaryotic small GTPase family (ArfRs) exclusive to the archaeal lineage from which eukaryotes emerged, namely, the Asgard archaea (Vargová et al., 2025; Zhu et al., 2025). The archaeal ArfRs share with their eukaryotic counterparts the diagnostic structural rearrangements of the N-terminal amphipathic helix in the GTP- vs. GDP-bound states, and GTP-bound ArfRs localize to membranes when heterologously expressed. This is the origin of all ARF family members.

**FIGURE 4:**
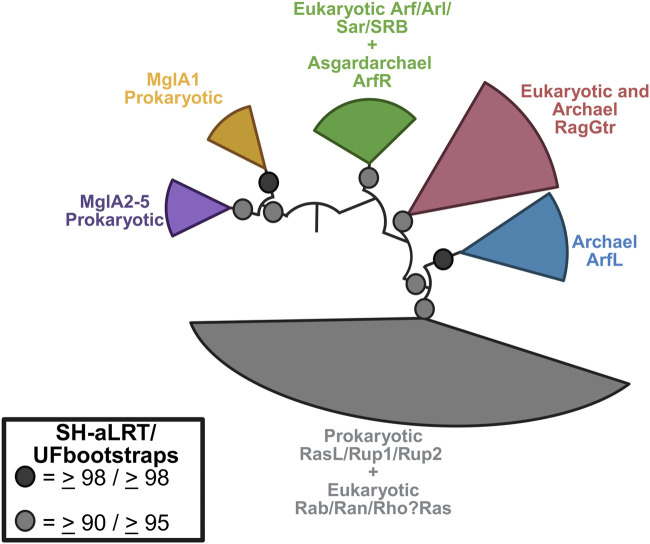
Phylogenetic relationship between the Arf family and other small GTPases. Eukaryotic Arf GTPases are related to Asgard archaeal ArfR (green) and are robustly separated from the Rag (maroon) and RasL (grey) families. The latter includes the Ras GTPases. Other archaeal GTPases families are also shown. Figure redrawn from Vargová et al. (2025) using BioRender.

Most importantly, the point here is that ARFs need to be considered separately from RAS and Gα. ArfRs and eukaryotic ARF families are robustly separated and thus evolutionarily distinct from RAS GTPases (Vargová et al., 2025) (Figure 4), which derive from the Asgard archaeal RasL, a different prokaryotic set of GTPases (Klinger et al., 2016). Although the heterotrimeric G proteins are predicted to have emerged in evolution from the ARF family (Anantharaman et al., 2011), their structure and activity are very different. In this review, we set aside the ARLs and SARs to focus on ARF1-6 as these are the best characterized in terms of their actions and mechanisms.

**FIGURE 5:**
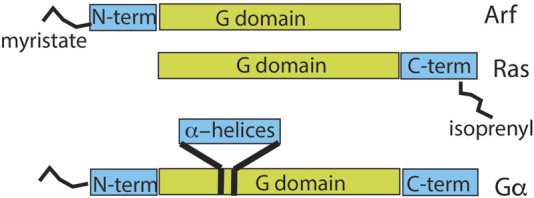
Comparison of domain structures of ARF, RAS, and heterotrimeric α-subunit. The relationship between the GTP-binding domain and other regions of the indicated GTP-binding proteins is shown. ARF has an N-terminal extension of 13–17 amino acids that is co-translationally myristoylated. RAS has a C-terminal extension of 22–24 amino acids that is isoprenylated and, in some family members, also palmitoylated. The α-subunit of G proteins has a 120-amino-acid insert in the G domain that forms an α-helical domain over the nucleotide-binding site. G-protein α-subunits also have 30–45 amino acid N-terminal and 20–25 amino acid C-terminal extensions, with the N-terminus myristoylated or palmitoylated in some gene products.

Second, although ARFs share a common GTP-binding ability or G-domain with RAS and G proteins, the primary structure differs in functionally important ways (Figure 5) (Der, 1989; Gilman, 1987; Hobbs et al., 2016; Sprang, 1997; Sztul et al., 2019). ARFs have an N-terminal extension of ∼13–17 residues from the G domain that is co-translationally modified by the covalent attachment of myristate to the N-terminal glycine residue and persists throughout the lifetime of the protein (Kahn et al., 2006; Sztul et al., 2019). In contrast, RAS proteins have a C-terminal extension that is isoprenylated and truncated by cleavage of the last three residues, leaving the isoprenylated cysteine as the C-terminal residue (Der, 1989; Khosravi-Far and Der, 1994; Nuevo-Tapioles and Philips, 2022; Shih and Weeks, 1984). Gα is similar to ARF in possessing an N-terminal extension that is lipid-modified but differs from ARFs in containing a ∼20 kDa insert within its G domain, which forms 6-α helices that cover the nucleotide-binding site. Furthermore, Gα has a C-terminal extension that interacts with GPCRs (Gilman, 1987; Sprang, 1997).

Third, the kinetics of GTP hydrolysis by ARF are distinct from those of other GTP-binding proteins. Although the intrinsic GTPase rates for both RAS and G proteins are slow (0.0003–0.002/sec and 0.01–0.1/sec) (Neal et al., 1988; Ross and Wilkie, 2000), they are undetectable in ARF. Using nuclear magnetic resonance (NMR) to monitor GTP bound to ARF, no hydrolysis was detected after 3 days. GAP-induced hydrolysis rates are similar among the three classes of GTPases, although they are somewhat faster for ARF, with maximum rates of 2–15/sec reported for RAS (Nixon et al., 1995; Phillips et al., 2003; Webb and Hunter, 1992), up to 25/sec for G proteins (Ross and Wilkie, 2000), and >50/sec for ARF (Jian et al., 2009; Luo et al., 2007).

**FIGURE 6:**
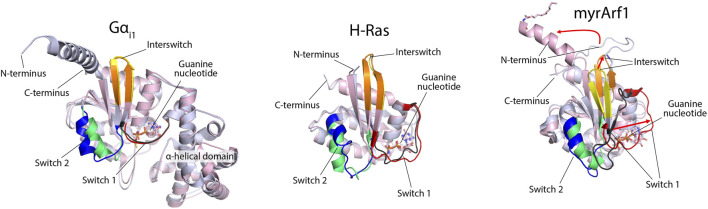
Structural differences between GDP- and GTP-bound forms of Gα_i1_, H-Ras, and myristoylated ARF1. ARF structures are reported by Liu et al. (2009), Liu et al. (2010) (PDB 2KSQ and 2K5U). Gα_i1_ structures are from Wall et al. (1995) and Coleman et al. (1994) (PDB 1GP2 and 1GIA) and include features that are absent in small G proteins, such as the α-helical domain. H-Ras structures are from Milburn et al. (1990) and Pai et al. (1990) (PDB 4Q21 and 5P21) and are missing the C-terminal extension. The GDP- and GTP-bound forms of the proteins are overlaid and are shown in light blue and light pink, respectively. Switch 1 is colored black in the GDP-bound form and red in the GTP-bound form for each protein. Switch 2 in the GDP-bound form is blue, and in the GTP-bound form, it is lime green. The interswitch is yellow in the GDP-bound form and orange in the GTP-bound form. For ARF, the three red arrows indicate the comparatively large (relative to Gα_i1_ or H-Ras) structural rearrangements necessary to cycle between GDP- and GTP-bound states.

Fourth, the conformational changes in response to nucleotide exchange are greater for ARFs than for heterotrimeric G protein α-subunits or other small G proteins such as RAS (Figure 6). The structural changes in ARF have been described as an “interswitch toggle” (Pasqualato et al., 2002), which is absent from the other two classes of GTPases. Here, we display the differences in yeast ARF1 in the GTP- and GDP-bound forms as an example (Figure 6) (Liu et al., 2009; 2010) and compare them to rat Gα subunit i1 (Gα_i1_) (Coleman et al., 1994; Wall et al., 1995) and human H-Ras (Milburn et al., 1990; Pai et al., 1990). The backbone residues of the GDP- and GTP-bound forms are shown in light blue and pink, respectively, in the cartoon (Figure 5). Two regions of GTPases that are sensitive to bound nucleotides are called switch 1 (Sw1) and switch 2 (Sw2), as indicated in the illustration. The interswitch domain includes the residues between the two switch regions, also indicated in the illustration. Sw1 in the GDP-bound form is shown in black, and that in the GTP-bound form is shown in red. As shown in Figure 5, the differences between the GDP- and GTP-bound forms of Gα_i1_ and H-Ras are subtle compared with those of ARF, in which Sw1 lies adjacent to the interswitch in the GDP-bound form and is displaced from the interswitch by approximately 15 Å in the GTP-bound form (red arrow, right side of the myrARF1 panel). Sw2 is shown in blue for the GDP-bound forms of the proteins and lime green for the GTP-bound forms. The differences in Sw2 in Gα_i1_ and H-Ras are more pronounced than those in Sw1 but are nonetheless less than those in ARF, where the α-helix is doubled in length and reoriented relative to the interswitch. ARF has additional structural differences between the GDP and GTP bound forms. The interswitch “toggles” from GDP-bound form in yellow to the GTP-bound form in orange (Pasqualato et al., 2002), with a greater than 10 Å difference in position and a shift in register relative to Sw1 and Sw2 (red arrow, middle of the myrARF1 panel). In the GDP-bound form, the interswitch forms part of a hydrophobic pocket in the protein, where the N-terminal myristate is accommodated. The N-terminal amino acid extension lies unstructured on the myristate. The position of the interswitch in the GTP form of ARF shortens the hydrophobic pocket so that it cannot accommodate the myristate. Consequently, the myristate is excluded from the protein to associate with lipid surfaces (red curved arrow, top of the myrARF1 panel). The N-terminal amino acid extension then forms an amphipathic helix that, together with the myristate, associates with the membrane phospholipid bilayer.

A fifth distinction between ARFs and other regulatory GTPases is the role of GTP hydrolysis in their functions. For RAS and G proteins, locking the proteins in the GTP-bound form increases the signals controlled by RAS or the G protein, e.g., the kinase cascade or cAMP generation (Campbell and Der, 2004; Clementi et al., 1990; Der, 1989; Gibbs et al., 1984; Hobbs et al., 2016; Landis et al., 1989; Raut et al., 2025; Sweet et al., 1984; Voyno-Yasenetskaya et al., 1994; Weinstein et al., 1991). In contrast, blocking the hydrolysis of GTP on ARF results in the loss of function of the regulated pathway. The requirement for GTP hydrolysis for ARF function is exemplified in its role in regulating COPI-dependent membrane traffic at the Golgi (Spang, 2002; Spang et al., 2010). In this paradigm, protein-coated vesicles form on a donor membrane and carry cargo to an acceptor membrane. ARF•GTP is necessary for the recruitment of coat proteins to the donor membrane to promote vesicle formation. Although the precise site and function of GTP hydrolysis have been debated, there is no debate that hydrolysis is also necessary for the successful transport of the cargo by the transport vesicle (Nie and Randazzo, 2006; Spang, 2002; Spang et al., 2010). Other ARF-regulated membrane traffic also depends on a cycle of GTP binding and hydrolysis. For instance, recycling of major histocompatibility complex I (MHC-1) to the cell surface is blocked by expression of a mutant of ARF6 that is deficient in GTP hydrolysis, with MHC-1 becoming trapped intracellularly (Klein et al., 2006). Similarly, phagocytosis in macrophages is blocked by expression of either a mutant of ARF6 that is deficient in GTP hydrolysis or a mutant that cannot bind GTP (Egami et al., 2015). Micropinocytosis through circular dorsal ruffles has also been found to be blocked by ARF1 and ARF5 mutants deficient in GTP binding and hydrolysis (Hasegawa et al., 2012).

Examples of ARF-dependent regulation of actin dynamics requiring a cycle of GTP binding and hydrolysis have also been described. Actin-rich membrane protrusions were driven by a fast-cycling mutant of ARF6 ([T157A]ARF6), which has low affinity for GDP and, consequently, reloads with GTP after GTP is hydrolyzed. GAPs can stimulate GTP hydrolysis by this mutant. In marked contrast, an ARF6 mutant deficient in GTP hydrolysis did not induce protrusions, consistent with the idea that both GTP binding and hydrolysis are necessary for this function (Klein et al., 2006; Santy, 2002). Other examples where GTP binding and hydrolysis by ARF are necessary for function include focal adhesions (FAs) and actin stress fibers (SFs). FAs are multiprotein complexes containing integrins, focal adhesion kinase, paxillin, and vinculin (Critchley et al., 1999; Kumari et al., 2024; Livne and Geiger, 2016). FAs connect to SFs, which are composed of alternating bundles of actin with non-muscle myosin 2 and actin with α-actinin. Emerging evidence indicates that both FAs and SFs can be coordinately controlled by ARFs (Yoon et al., 2025). Reduced ARF5 and ARF5 mutant expressions (accomplished by treatment of cells with small interfering RNA) with either decreased GTP binding ([T31N]ARF5) or decreased GAP-dependent GTPase activity [(I46D)ARF5 (Luo et al., 2005; Yoon et al., 2025)] reduced the number of FAs and SFs, consistent with the interpretation that both GTP binding and hydrolysis by ARF5 are necessary for the maintenance of these structures (summary of differences in Table 1).

**TABLE 1:** Comparison of ARF, RAS, and Gα.

	ARF	RAS	Gα
Size (kDa)	∼21	∼21	∼45
Additions to G domain	N-terminal extension	C-terminal extension	N-terminal extension Insert in G domain C-terminal extension
Lipid modifications	Myristoylation	Isoprenylation Palmitoylation	Myristoylation Palmitoylation
Intrinsic GTPase	None	∼0.002/sec	∼0.1 s
Effect of blocking GTP hydrolysis on the effect of GTPase	Decrease	Increase	Increase
Number of GAPs	>28	9	>20

References for table (Bos et al., 2007; Gilman, 1987; Kahn et al., 2008; Kahn et al., 2006; Ross and Wilkie, 2000; Saraste et al., 1990; Sprang, 1997; Sztul et al., 2019; Vetter and Wittinghofer, 1999; Wall et al., 1995; Willars, 2006; Zheng et al., 1999).

### 2.3 Chapter 3. ARFs may be better understood in a P loop NTPase framework

As described in the prologue (section 1), ARF, RAS, and G proteins are P loop NTPases. The ancestor of the larger family of P loop NTPases likely evolved before the last universal common ancestor and evolved into multiple families (Leipe et al., 2003; Orengo and Thornton, 2005; Saraste et al., 1990; Shalaeva et al., 2018; Vetter and Wittinghofer, 1999; Walker et al., 1982). A non-inclusive list of protein families includes the ARF family, signal recognition particle receptor β, the RAS superfamily, G proteins, myosin, kinesin, dynein, dynamin, elongation factors, adenylate kinase, septins, nucleoside diphosphate kinase, and F1–ATPase. The function of these proteins is varied and not restricted to signaling and nucleotide metabolism. Since GTP binding and hydrolysis are both necessary for ARF function, we consider how other P-loop NTPases integrate nucleotide binding and hydrolysis to define ARF context-specific function. Toward that end, we present three examples: myosin, elongation factors, and SRPRβ.

**FIGURE 7:**
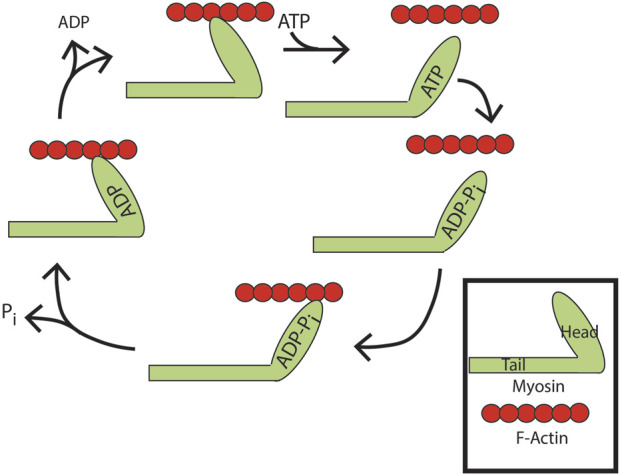
ATP binding and hydrolysis cycle of myosin. Apo-myosin binds tightly to actin filaments until ATP binding to myosin induces its release. ATP is subsequently hydrolyzed. Both products, ADP and orthophosphate, remain associated with myosin. In this state, myosin rebinds actin with evacuation of one reaction product, orthophosphate. This results in a conformational change in the lever arm of myosin, pulling the actin filament. ADP is then released leaving apo-myosin tightly bound to the actin filament, with net movement along the filament.

Example 1 : Myosin, a motor protein (Figure 7, note that the diagram is simplified to show just one member of the myosin dimer for the sake of clarity) (Batters et al., 2014; Goody and Hofmann-Goody, 2002; Heissler and Sellers, 2016; Pecci et al., 2018; Sellers, 2000; Vicente-Manzanares et al., 2009): Movement of myosin on filamentous actin (F-actin) is achieved using energy from ATP hydrolysis. Myosin without nucleotide binds to actin filaments. ATP binding displaces myosin from F-actin. ATP is hydrolyzed, but both products, ADP and orthophosphate, remain associated with myosin. In this state, myosin binds to actin with the release of one reaction product, orthophosphate. This results in a conformational change in the lever arm of myosin, pulling the actin filament. ADP is then released. Actin accelerates ATP hydrolysis by increasing the rate of product (ortho-phosphate) release. From another perspective, actin is an ATP hydrolysis-activating protein.

**FIGURE 8:**
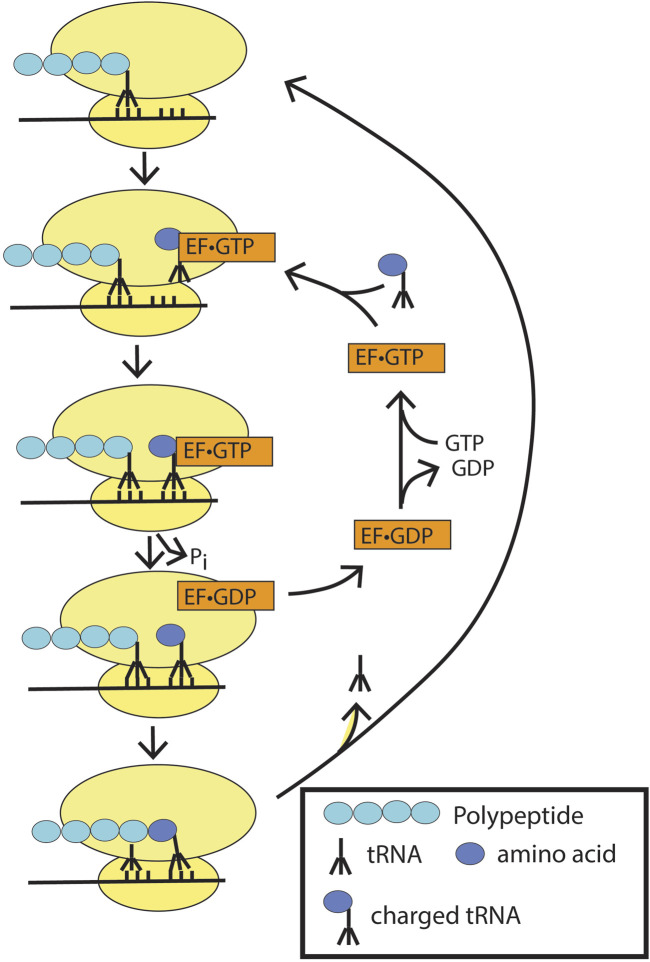
Elongation factor-Tu (EF-Tu) in peptide synthesis. Ef-TU•GTP bound to charged tRNA binds to the peptidyl site on the ribosome. If the codon matches, GTP hydrolysis is triggered with the release of the charged tRNA into the site with the matched codon. EF-Tu•GDP dissociates from the ribosome, and the peptidyl bond is formed to extend the polypeptide chain. The ribosome is depicted as two yellow ovals, and the mRNA as a black line through the ribosomes.

Example 2 Elongation factor-Tu (EF-Tu), proofreading during protein translation (Figure 8) (Dever et al., 2018; Pape et al., 1998).

EF-Tu functions with tRNA to ensure incorporation of the correct amino acids during polypeptide translation on ribosomes (Figure 8). On a ribosome, the growing peptide chain is anchored through a tRNA that matches the three-nucleotide codon in the messenger RNA in a site called the peptidyl-tRNA site (P site). An adjacent site on the ribosome contains codon 3′ to the codon in the P site in the mRNA. This is called the aminoacyl-tRNA or A-site. EF-Tu•GTP linked to a tRNA charged with an amino acid is delivered to the A-site. If the codon matches the charged tRNA linked to EF-Tu, GTP hydrolysis is triggered, which is accompanied by a conformational change. The amino group from the aminoacyl site attacks the carbonyl carbon of the peptide in the peptidyl-tRNA site, forming a peptide bond and breaking the bond between the tRNA and the peptide that had been in the P site, leaving the peptide covalently linked to the tRNA with the newly added residue. There is then a shift in register, with the transfer of the linked tRNA from the A site to the P site. The tRNA that was displaced from the peptide on bond formation moves to the exit site (not depicted in the figure). Elongation factor then dissociates from the ribosome to complete the cycle. This cycle of GTP binding and hydrolysis ensures the high fidelity required for protein synthesis. Previous studies have shown that GTP hydrolysis is essential for ribosome function, with failure to hydrolyze GTP resulting in defective A-site binding (Kaziro, 1978).

**FIGURE 9:**
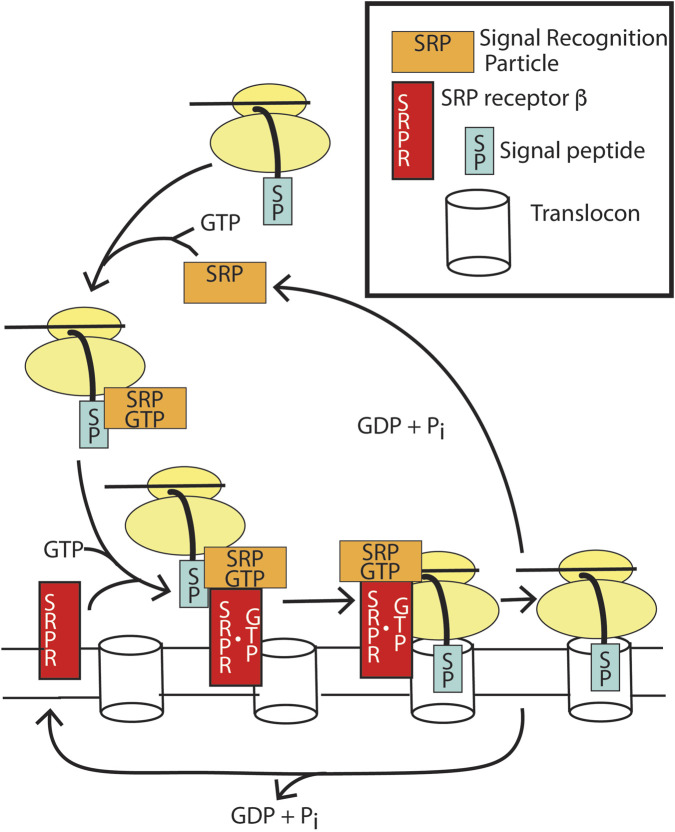
Function of SRP and SRPRβ for translocation of transmembrane and secreted proteins. SRP•GTP binds to the emerging signal peptide during translation of membrane and secreted proteins, which pauses translation. SRP•GTP docks with apo SRPRβ, which docks to the translocon, resulting in GTP binding to SRPRβ. The dimer of SRP •GTP:SRPRβ•GTP results in the transfer of the signal peptide into the translocon. GTP hydrolysis by both SRP and SRPRβ results in the release of apoSRP and SRPRβ and, consequently, release of the translational pause.

Example 3 The SRP receptor β targets translation of the transmembrane and secreted proteins to the translocon (Figure 9) (Rapoport, 1986; Walter and Lingappa, 1986).

Transmembrane and secreted proteins are inserted into and transferred through, respectively, the ER membrane during translation. Ribosomes are targeted to membranes containing a protein pore, termed the translocon, by the actions of two GTP binding proteins: signal recognition particle (SRP) and the signal recognition particle receptor (SRPRβ). A 15–30 amino stretch of amino acids at the N-termini of transmembrane and secreted proteins, called the signal peptide, is recognized by SRP, resulting in a block in translation. SRP•GTP then targets the ribosome to the ER membrane by binding to a second GTP-binding protein, SRP receptor β, promoting GTP binding and stabilizing the complex. The translocon binds to the SRP-GTP: SRPR-GTP complex, initiating transfer of the signal peptide to the translocon. Once the signal peptide and ribosome are docked with the translocon, SRP and SRPR induce reciprocal GTP hydrolysis. GTP hydrolysis triggers SRP dissociation from the ribosome and SRPR dissociation from the translocon, allowing protein translation to resume. In contrast to the regulatory GTPases described above, GTP hydrolysis is achieved not through a GAP but instead through the combined actions of the two GTP-binding proteins, each acting as a GAP for the other (Gasper et al., 2009).

These three examples, along with ARF, diverge from the classic GTPase signaling paradigm as their function requires a cycle of nucleotide binding and hydrolysis, and the lack of either binding or hydrolysis results in blockade of the pathway.

### 2.4 Chapter 4. GTPase-activating proteins are integral to ARF function

Myosin, EF-Tu, and SRPRβ are only three examples of how nucleotide binding and hydrolysis can be used to direct cellular functions rather than transduce signals. In each case, a second protein that induces GTP hydrolysis is necessary for the function. In the case of ARF, hydrolysis of GTP is necessary for function, but ARF has no detectable intrinsic GTPase activity. Consequently, the GAPs are necessary for function, not, as described in the current paradigm, to terminate ARF function. With this consideration, we propose a different perspective and possible lens for the role of ARFGAPs as mediators of ARF function.

The first speculation that proteins might serve as both effectors and GAPs arose in the early 1990s for G proteins and RAS (Berstein et al., 1992; Tocque et al., 1997). The concept of a GAP being an effector for ARFs has been more recently discussed (East and Kahn, 2011; Spang et al., 2010; Yoon et al., 2025). The possibility for effector functions for ARF GAP was discussed as early as 1994 (Randazzo and Kahn, 1994). The first evidence for ARF GAPs mediating ARF function, thus being key components in the pathway and not terminators of the ARF signal, came out of a screen in the yeast *S. cerevisiae* for suppressors of a temperature-sensitive hypomorphic point mutant of ARF1 (Zhang et al., 1998). The screen yielded only four genes capable of rescuing the growth of yeast with ARF insufficiency when overexpressed. Each encoded a yeast ARF GAP (as made evident by the presence of the ARF GAP domain in each). If the function of GAPs is to terminate an ARF signal, then increasing ARF GAP levels or activity should exacerbate ARF insufficiency. The result, contrary to the prediction of the signaling paradigm, supports the hypothesis that ARF GAPs are components of the ARF effector machinery. A plausible mechanism for the effect of ARF GAPs in yeast was later supported by the finding that the ARF GAPs bind to SNARES, a component of vesicles necessary for fusion with an acceptor membrane, inducing a conformational change that results in increased affinity of the SNARES for coat proteins (Rein et al., 2002; Schindler et al., 2009; Schindler and Spang, 2007). One important function of ARF is sorting cargo into transport vesicles. One interpretation of the suppression screen yielding the four ARF GAPs is that, at high concentrations, ARF GAPs could have driven sorting of SNAREs into protein-coated transport vesicles independent of ARF, promoting cell survival.

**FIGURE 10:**
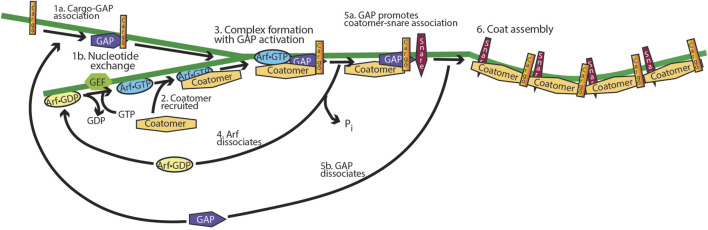
Model for the function of ARF GAP1/2 and ARF in cargo sorting into transport vesicles. ARF•GTP binds to and recruits vesicle coat proteins to a donor membrane-bound organelle, and the ARF GAP binds to cargo. A complex is then formed which includes coat protein, ARF•GTP, ARF GAP, and cargo. The GAP triggers GTP hydrolysis, leading to ARF dissociation and cargo binding to the coat. The GAP binds to snare proteins, promoting coat binding with dissociation of the ARF GAP, followed by coat polymerization. The product is a cargo-laden, protein-coated vesicle containing the fusion machinery necessary for delivering the cargo to an acceptor membrane.

The idea of an ARF GAP controlling cargo sorting during the assembly of vesicle coats is also supported for mammalian cells (Figure 10) (Shiba and Randazzo, 2012; Spang et al., 2010). Both in reconstitution systems, in which Golgi membranes were incubated with soluble fractions from cells, and in tissue culture, blocking GTP hydrolysis reduced the generation of cargo-laden vesicles (Bremser et al., 1999; Kartberg et al., 2010; Lanoix et al., 1999; Lanoix et al., 2001; Nickel et al., 1998; Pepperkok et al., 2000; Weiss and Nilsson, 2003). When the expressions of ARF GAP1, ARF GAP2, and ARF GAP3 were reduced together, proteins that were usually resident in the cis Golgi compartment were found in the ER–Golgi intermediate compartment, similar to reducing the expression of the coat protein coatomer (Saitoh et al., 2009). Taken together, a plausible hypothesis is that ARF GAP1, 2, and 3 function to sort cargo into vesicles and promote assembly of fusion-competent vesicles, as illustrated in Figure 10. An important distinction from other small GTPases and G proteins is that ARF in the GTP form blocks sorting into vesicles. Thus, sorting is considered the signal (which is not precise), and therefore, ARF•GTP blocks the signal. Based on these results, one would hypothesize that ARF recruits coat protein and ARF GAP, if bound to the appropriate cargo, associates with the ARF–coatomer complex. This would trigger GTP hydrolysis with transfer of the cargo to the coat protein, followed by coat assembly. Interaction with SNARES, increasing affinity for coat proteins, would also contribute to coat assembly. Without the GAPs to promote the formation of transport vesicles, the proteins are trapped in the ER–Golgi intermediate compartment.

**FIGURE 11:**
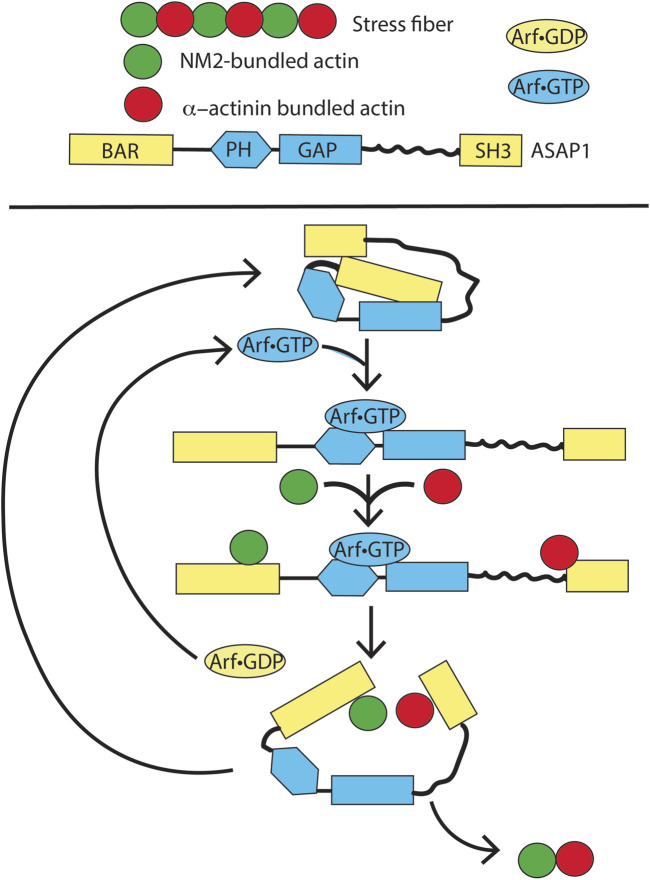
Speculation about the function of ARF and an ARF GAP, ASAP1, in the formation of stress fibers. In a ground state, ASAP1 is folded through two hinges. On ARF•GTP binding, the protein unfolds revealing binding sites for F-actin bundled with NM2 and α-actinin. GTP hydrolysis leads to ARF•GDP dissociation and partial refolding of ASAP1. The partial refolding drives fusion of the two types of F-actin bundles, thereby assembling stress fibers. This model is currently being tested *in vitro*.

In this same theme of ARF GAPs mediating ARF activity, recent evidence supports the idea that the ARF GAP ASAP1 mediates the effects of ARF on SFs (Figure 11). ASAP1 is composed of BAR, PH, ARF GAP, ankyrin repeat, proline-rich, E/DLPPKP repeat, and SH3 domains. ASAP1 folds on two hinges in the ground state, with the BAR and PH domains contacting the ARF GAP domain and the C-terminus contacting the PH and ARF GAP domains. Loss of ASAP1 results in a loss of SFs (Chen et al., 2016; Gasilina et al., 2019; Gasilina et al., 2022). SFs are composed of strings of alternating non-muscle myosin 2 (NM2) and α-actinin-bundled actin, appearing as beads on a string (see Figure 11) (Lehtimäki et al., 2017; Livne and Geiger, 2016; Tojkander et al., 2012). ASAP1 binds to actin and NM2 through its BAR domain (Chen et al., 2020; Chen et al., 2016; Gasilina et al., 2019). Based on preliminary data, ASAP1 also binds to α-actinin, with ASAP1 partially overlapping both NM2 and α-actinin in SFs to bridge the two structures. ASAP1-dependent maintenance of SFs requires both binding actin and the ARF GAP domain (Yoon et al., 2025). Together with the findings that SF formation also depends on ARF5•GTP and GTP hydrolysis and that the ARF GAP domain inhibits actin and NM2 binding to the BAR domain, these data led us to propose the hypothesis that ASAP1 mediates the assembly of SFs. In this hypothesis, ASAP1, unfolded by binding to ARF5•GTP, binds to NM2 and α-actinin-bundled actin. GTP hydrolysis and release of ARF lead to partial refolding of ASAP1, resulting in fusion of the two bundles. The fused bundles are released, and ASAP1 returns to the ground state. Although this hypothesis is actively being tested, we present it here to serve as a model for a different conceptual framework to examine the role of an ARF GAP in mediating ARF functions.

From these examples, we propose a new paradigm in which the GAPs do not control ARF•GTP levels but instead specify and differentially carry out ARF function. This perspective on ARFs may provide an opportunity to advance our understanding of the mechanism of ARF action and could explain observations such as the localization of multiple ARF GAPs with similar ARF specificities to the same subcellular compartment. Each ARF GAP could carry out a specific biological function in response to ARF•GTP to achieve a multifaceted response.

It is worth noting that the idea that ARF GAPs enable ARF GTPases to have context-specific functions is consistent with the fact that ARFGAP diversity outstrips ARF diversity at various points of evolution (as has been reconstructed through a number of phylogenetic studies) (Jackson et al., 2023). Although much remains unclear concerning ARF GAP biology, including critical details such as which GAPs act on which ARFs (much less which ARLs), a pattern nonetheless emerges from the known data. At the earliest reconstruction point for the eukaryotic ARF regulatory system, the last eukaryotic common ancestor, there are only ARF1 and ARF6, but at least seven ancestral ARFGAPs (Jackson et al., 2023). In humans, there are 5 ARFs and approximately 30 ARF GAPs. The inference, then, is that the ARF GAPs diversified more extensively during the period, leading to the eukaryotic ancestor, consistent with the differentiation of function being driven by ARF GAPs mediating the activity of only a few ARFs.

## 3 Epilogue

**FIGURE 12:**
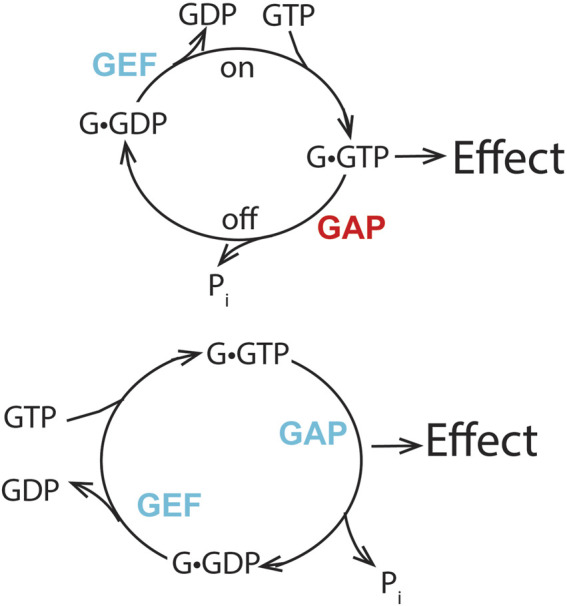
Distinguishing features of two mechanisms of GTPase action. The prevailing model for signaling GTPases is shown in the upper panel. The GTPase•GTP complex binds to an effector to bring about an effect. Blocking formation of the GTPase•GTP by preventing exchange or accelerating GTP hydrolysis reduces the effect. Increasing the rate of GTPase•GTP formation or blocking the hydrolysis of GTP increases the signal. Thus, the GEF and GAP have opposing functions. The lower panel depicts our proposal for ARFs. The transition of ARF•GTP to ARF•GDP results in an effect. Blocking either exchange or GTP hydrolysis reduces or blocks the effect. Increasing the rate of the cycle may increase the effect. Thus, the GEF and GAP have collaborative function. Blue lettering indicates that the GAP or GEF increases the effect of Arf, while red lettering indicates that the GAP decreases the effect of Arf in the hypothesis presented in the upper panel.

In this review, we describe the importance of the GTP binding and hydrolysis cycle and the role(s) of ARF GAPs for mediating ARF biological functions. The importance of the binding/hydrolysis cycle for achieving ARF activity, in addition to other biochemical properties, distinguishes ARF from signaling GTPases such as RAS and G proteins. We have proposed a different way of thinking about ARF GAPs, in which the ARF GAPs mediate the effects of ARF, in sharp contrast to the prevailing paradigm of the GAPs functioning to downregulate signaling GTPases such as RAS (Figure 12).

Discriminating between the two mechanisms remains to be directly tested; however, the available literature, as described above, supports the idea. We are not aware of the literature directly refuting the hypothesis that GAPs mediate nucleotide cycling-dependent ARF function. The argument that ARF GAPs terminate ARF signals is based primarily on the fact that the GAPs can hydrolyze GTP bound to ARF, coupled with the assumption that ARFs are signaling GTPases. Rescue of exchange factor deletions with fast cycling mutants, but not GTPase-deficient mutants, is consistent with this rather than the ARF signaling model. When GTPase-deficient mutants have been used to rescue an exchange factor deficiency, the authors have noted high toxicity and rescue only at low expression levels of GTPase-deficient mutants (as the experiments were performed in ARF wild-type background cells).

Further understanding of the functions of the diverse domains found in ARF GAPs will enable direct testing of the hypothesis that ARF GAPs mediate ARF function dependent on both ARF•GTP binding to the GAP and GTP hydrolysis. The ARF GAPs containing the PH–ARF GAP–ankyrin repeat tandem have additional domains with both known binding partners and functions, providing relatively straightforward models for testing. Along with the data presented in this review, previous studies that provide evidence in support of this hypothesis include ARF GAPs that function with coat proteins (Lanoix et al., 1999; Nickel et al., 1998; Nie and Randazzo, 2006; Pepperkok et al., 2000; Saitoh et al., 2009; Schindler et al., 2009; Spang et al., 2010; Weiss and Nilsson, 2003). Additional testing of the hypothesis is, at least, in part, dependent on advances in solving the structures of the protomeric and polymerized forms of the coat proteins, both with ARF and GAPs bound. Examining the data from the perspective of the GAPs being a subunit of coat protomers may provide additional insights; after all, the SAR1-dependent coat has a subunit that is a GAP for SAR.

This paper focused on the ARF subfamily of ARF GTPases. Other ARF family members likely function outside the signaling paradigm. ARL2, ARL3, and ARL13B are known to use distinct mechanisms of activation and inactivation. For example, ARL2 controls tubulin folding, and the close relative of ARF, SRPRβ, has a well-defined function outside both the paradigm for GTPase signaling and the mechanism proposed here for ARFs. Thus, exploration into ARF function should not be constrained by the signaling paradigm. Simultaneously, canonical signaling should not be ruled out for all ARFs; heterotrimeric G proteins evolved from ARFs (Anantharaman et al., 2011), so there are some ARF family members that likely function as signaling GTPases. Among the ARFs considered in this paper, ARF6 is predicted to have both a signaling and a non-signaling function. Both upstream signaling elements (e.g., insulin, IGF-1 receptors, TrkC, and somatostatin receptors) that affect exchange factors, including EFA6A and cytohesins, and canonical effectors (e.g., cytohesins, which act as both effectors and exchange factors, phospholipase D, and phosphoinositide 4-phosphate 5-kinase) have been identified for ARF6 (Clodi et al., 1998; Esteban et al., 2006; Grodnitzky et al., 2007; Kawaguchi et al., 2014; Klarlund et al., 1997; Lim et al., 2010; Malaby et al., 2013; Hafner et al., 2006). Yet, at least some of ARF6 functions are linked to GTP binding and hydrolysis (Klein et al., 2006; Santy, 2002). Re-examination of effects through ARF triggered by receptor occupancy is likely to be valuable, particularly with some recent evidence that proteins once considered ARF effectors are more efficiently activated by ARLs (Li et al., 2022).

As we work toward developing models of the ARF GTPase function, the path forward requires a detailed study of compartment and context specificity. Because ARF GTPases play a central role in critical cellular functions, their activities must be tightly coordinated to ensure that each function is executed at the correct time. Increasing evidence indicates that ARF GAPs are critical determinants of ARF GTPase functional specificity. Important future directions will require interdisciplinary approaches, combining fundamental biochemical approaches to systematically assess which ARFs act through which GAPs, with cell biological strategies to determine which GAPs coordinate which pathway(s) from which compartment(s). We can extend this analysis by defining the specific signaling contexts in which these ARF GAPs act. How do such features as cell cycle status, metabolic status, cell type-specific signaling cues, and disease state contribute to determining which ARFs and which GAPs are active in different contexts? Understanding how the fundamental biochemistry of ARFs and their GAPs is modulated by biological context will help elucidate critical aspects of GTPase biology that remain largely unexplored.

So, rather than being merely misunderstood, ARFs remain incompletely understood. Efforts to elucidate the biochemistry and biology of this ancient protein family are only beginning.
